# The negative NK cell maturation checkpoint Foxo1

**DOI:** 10.18632/oncotarget.6109

**Published:** 2015-10-13

**Authors:** Youcai Deng, Jianhua Yu

**Affiliations:** Division of Hematology, Department of Internal Medicine, College of Medicine, and Comprehensive Cancer Center, The Ohio State University, Columbus, Ohio, USA

**Keywords:** natural killer cells, maturation, checkpoint, Foxo1, Immunology and Microbiology Section, Immunity, Immune response

Natural killer (NK) cells, first introduced into the immunological lexicon around forty years ago, constitute the innate immunity arm of the lymphoid lineage based on their capacity to lyse abnormal or infected cells quickly and directly without prior sensitization. This ability makes NK cells critical in both anti-viral and anti-tumor immunity, where foreign antigens may be absent or suppressed. While the regulatory mechanisms of NK cell development and maturation have historically been less understood versus other immune subsets, great advances have recently been made in this field. For example, the early stages of NK cell development are now known to be regulated by Id2, E proteins, STAT5, IRF-2, Tox, Ets1, Nfil3, and among others, while T-bet, Eomes, Aiolos and Blimp-1 play critical roles in development and/or maturation [1]. Additional genes found to promote NK cell late-stage maturation include mTOR [2], SHIP1, Notch [1], and Runx3 [1]. However, negative regulators in the process of NK cell development are still largely unknown.

Previous findings showed that the transcription factor Foxo1 plays critical roles in regulating the development of common lymphoid progenitors as well as T and B cells in a highly cell- and context-specific manner. In a recent issue of *Immunity*, we defined Foxo1 as a negative checkpoint regulator of NK cell late-stage maturation and effector function [3]. Mechanistically, we showed that in mice, Foxo1 is recruited by Sp1 to the promoter of Tbx21 and inhibits its expression. This finding solves a puzzle in the field of Foxo1 research, as Foxo1 was shown to inhibit the expression of many target genes despite its inability to directly bind to putative Foxo1 sites in the promoter of these genes. In our study, Foxo1 deficiency was induced using Ncr1 (NKp46)-driven Cre recombinase (Ncr1i^Cre^) in mice. While this allowed us to study the role of Foxo1 in late stage of the NK cell lineage, we were not able to determine whether Foxo1 is also involved in early-stage NK cell development prior to the expression of Ncr1.

Foxo1 is downstream of the PI3K/Akt pathway. Both the regulatory subunit (p85) and the catalytic subunit (p110) of the class I PI3Ks have been shown to positively regulate NK cell terminal maturation [2]. mTOR is an important downstream component of the PI3K signaling pathway [2]. mTOR, PDK1 (a kinase upstream of mTOR), and E4BP4 (a basic leucine zipper transcription factor downstream of mTOR) have recently been demonstrated to be positively control NK cell development and maturation [2]. It can be speculated that other components, in addition to the above factors and Foxo1, in the mTOR signaling pathway and/or in the PI3K signaling pathway in general might also be involved in NK cell development and/or maturation.

Accumulating evidence supports the idea that NK cells can mount a form of antigen-specific immunologic memory, undergo a clonal-like expansion during cytomegalovirus infection, generate long-lived progeny, and mediate higher secondary responses against previously encountered cytomegalovirus, especially in murine models. NK cell memory can also be identified after hapten exposure or following cytokine pre-activation. Many genetic and epigenetic factors have been shown to regulate NK cell memory. For example, recent genome-wide expression studies have discovered novel pathways involved in regulating antigen-specific effector and memory NK cell responses such as the Zbtb32, microRNA-155, Noxa and SOCS1 networks [4]. The most recent studies reveal that epigenetic modification of the genome and silencing of PLZF are involved in NK cell memory after cytomegalovirus infection in humans [4]. However, the underlying mechanisms for sustaining NK cell memory remain elusive. Memory NK cells show a characteristic immunophenotype including higher expression levels of several surface markers, such as KLRG1, CD43, and Ly6C, but lower expression of CD62L and CD27 [5]. Our current study found that conditional depletion of Foxo1 in murine NK cells leads to an increase in the KLRG1^hi^CD43^hi^ NK cell population and lower expression of CD62L at the resting state [3]. These findings suggest Foxo1 might also regulate NK cell memory, although additional experiments are required to determine this.

NK cells are capable of trafficking into different tissues and microenvironments infiltrated by pathogens and tumors. NK cells egress from the bone marrow to various peripheral organs in the steady state, a process controlled by a variety of adhesion molecules such as integrins, selectins, and chemokine receptors and their corresponding ligands [6]. NK cells at different developmental stages express a distinct repertoire of adhesion molecules, and can therefore be recruited to different sites of the body, including lymphoid and non-lymphoid tissues. After arriving at a specific site with local microenvironmental signals, NK cells undergo further differentiation into unique tissue-specific NK cells [7]. L-selectin (CD62L) is expressed on NK cells, which is required for their entry into lymph nodes. PTEN, a component in the PI3K signaling pathway, has been shown to regulate NK cell trafficking in mice [8]. In our recent manuscript, we also found that Foxo1 deficiency in NK cells results in a decreased NK cell numbers in peripheral lymph nodes, correlating with CD62L protein down-regulation. This indicates that Foxo1 promotes NK cell homing to lymph nodes by upregulating CD62L expression [3]. Additional work is underway to investigate whether Foxo1 controls NK cell trafficking into microenvironments where pathogens or tumor cells reside.

In conclusion, Foxo1 serves as a key negative checkpoint regulator at least for maturation, but may also exhibit other critical functions in NK cells. Further studies will be necessary to fully define the complex role of Foxo1 in NK cells.

## References

[1] Sun JC (2015). Curr Top Microbiol Immunol.

[2] Ali AK (2015). Front Immunol.

[3] Deng Y (2015). Immunity.

[4] Beaulieu AM (2015). Adv Exp Med Biol.

[5] Sun JC (2011). J Immunol.

[6] Peng H (2014). Clin Rev Allerg Immu.

[7] Yu J (2013). Trends Immunol.

[8] Leong JW (2015). P Natl Acad Sci Usa.

